# Are we drawing the right conclusions from randomised placebo-controlled trials? A post-hoc analysis of data from a randomised controlled trial

**DOI:** 10.1186/1471-2288-9-41

**Published:** 2009-06-23

**Authors:** M Diana van Die, Kerry M Bone, Henry G Burger, Helena J Teede

**Affiliations:** 1 School of Health Sciences, RMIT University, Bundoora, Victoria, Australia; 2 MediHerb Australia Pty Ltd, Warwick, Queensland, Australia; 3 School of Health, University of New England, Armidale, New South Wales, Australia; 4 Prince Henry's Institute of Medical Research, Clayton, Victoria, Australia; 5 Jean Hailes Foundation for Women's Health, Clayton, Victoria, Australia; 6 School of Public Health, Monash University, Clayton, Victoria, Australia

## Abstract

**Background:**

Assumptions underlying placebo controlled trials include that the placebo effect impacts on all study arms equally, and that treatment effects are additional to the placebo effect. However, these assumptions have recently been challenged, and different mechanisms may potentially be operating in the placebo and treatment arms. The objective of the current study was to explore the nature of placebo versus pharmacological effects by comparing predictors of the placebo response with predictors of the treatment response in a randomised, placebo-controlled trial of a phytotherapeutic combination for the treatment of menopausal symptoms. A substantial placebo response was observed but no significant difference in efficacy between the two arms.

**Methods:**

A *post hoc *analysis was conducted on data from 93 participants who completed this previously published study. Variables at baseline were investigated as potential predictors of the response on any of the endpoints of flushing, overall menopausal symptoms and depression. Focused tests were conducted using hierarchical linear regression analyses. Based on these findings, analyses were conducted for both groups separately. These findings are discussed in relation to existing literature on placebo effects.

**Results:**

Distinct differences in predictors were observed between the placebo and active groups. A significant difference was found for study entry anxiety, and Greene Climacteric Scale (GCS) scores, on all three endpoints. Attitude to menopause was found to differ significantly between the two groups for GCS scores. Examination of the individual arms found anxiety at study entry to predict placebo response on all three outcome measures individually. In contrast, *low *anxiety was significantly associated with improvement in the active treatment group. None of the variables found to predict the placebo response was relevant to the treatment arm.

**Conclusion:**

This study was a *post hoc *analysis of predictors of the placebo versus treatment response. Whilst this study does not explore neurobiological mechanisms, these observations are consistent with the hypotheses that 'drug' effects and placebo effects are not necessarily additive, and that mutually exclusive mechanisms may be operating in the two arms. The need for more research in the area of mechanisms and mediators of placebo versus active responses is supported.

**Trial Registration:**

International Clinical Trials Registry ISRCTN98972974.

## Background

The placebo-controlled trial is considered the gold standard among clinical research designs. The challenge of rigorous scientific research is to accurately determine the specific effect of an intervention over and above the placebo effect, (also referred to as 'non-specific effects', or 'context effects'). Failure to do so may result in the rejection of the intervention as ineffective as a potential treatment, as any benefits are ascribed to a placebo effect. We question this approach and suggest that inappropriate rejection of potentially viable treatments may be occurring.

**Figure 1:**
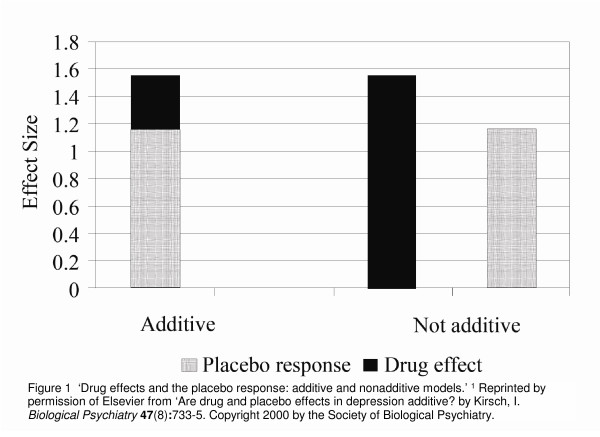
**'Drug effects and the placebo response: additive and nonadditive models'**[1]. Reprinted by permission of Elsevier from 'Are drug and placebo effects in depression additive? by Kirsch, I. *Biological Psychiatry ***47**(8):733–5. Copyright 2000 by the Society of Biological Psychiatry.

The underlying assumption of placebo-controlled trials is that, for participants blinded as to their group assignment, the placebo component affects all arms equally, with the specific effect of the active intervention/s being additional to the placebo effect in the intervention arm/s. This has been termed the 'additivity' of effects. However, this assumption has recently been challenged. It has been argued by Kirsch and colleagues [1] that it is not a logical necessity for the effects of the active treatment to be additive, or composed of the two components – the placebo effect and the specific treatment effect (see Figure 1). In support of their position they suggest that, if drug effects and placebo effects are additive, then the pharmacological effect of antidepressant drugs must be quite small [1], since meta-analyses of antidepressant drugs have found that 65% – 80% of the response to the drug is duplicated in the placebo arm, including in long-term maintenance studies [2-4]. They thus proposed that the effects may be non-additive or only partially additive [1], suggesting different underlying mechanisms may be operating in the placebo and pharmacological treatment arms.

One obvious conclusion from this observation is that antidepressant medication *does*, in fact, exert a very small pharmacological effect. Another possible explanation that has been proposed is that different neurobiological mechanisms may be operating in the two arms. The placebo may induce effects via psychological mechanisms only In the absence of a pharmacological effect, while the active treatment works through pharmacological mechanisms alone [5]. Some support for this hypothesis is derived from brain-imaging studies of depressed subjects, showing that placebo and active treatments induce quite different changes in brain function, despite exerting similar benefits [6-8]. Similarly, neurophysiological research on analgesia has suggested that expectation pathways, rather than pain pathways, may be stimulated by placebo treatment [9]. Expectation of reward has been shown to be at least partly mediated by the dopaminergic system [10-13], stimulation of which may be activated by the brain opioid system [9,14,15]. There is evidence that both endogenous opioids [16,17] and placebo-induced dopamine release may be relevant to the placebo effect [18-20]. Participant-related factors identified that may be responsible for the effects produced by placebos include Pavlovian conditioning resulting from prior exposure to the therapeutic intervention, and the expectation of reward (clinical benefit, in this case) [21].

In this setting, the current study analysed data from a previously published double-blind, placebo-controlled, RCT that had found no significant effect over placebo on any of the endpoints [22] for the study treatment, which was therefore concluded to exert no more than placebo effects. A comparison was made between predictors of the response in the placebo arm and predictors of the response to the active treatment. It was hypothesised that, if the additivitiy assumption is correct, then the same variables would predict the response in both groups.

## Methods

This study was a *post hoc *analysis of data from an investigation of 93 participants who completed a randomised, placebo-controlled, double-blind trial. We have previously published the outcome data on efficacy of the therapy [22] and predictors of the placebo response in the placebo group only [23]. Here we extend this evaluation to examine whether these predictors are also relevant to the treatment arm, and include data from all study participants. The original RCT had investigated the effect of a phytotherapeutic combination, consisting of the herbs *Hypericum perforatum *and *Vitex agnus-castus*, for menopausal symptoms in late-perimenopausal and postmenopausal women [22]. Following entry to the study, a two-week non-treatment run-in preceded the 16-week treatment phase. Endpoints included flushing, overall menopausal symptoms measured on the Greene climacteric scale (GCS) and depressive symptoms measured on the Hamilton Depression Inventory (HDI), both well-validated widely available tools. The trial was approved by the Human Research Ethics Committee at Royal Melbourne Institute of Technology University.

### Study intervention

As previously described [22], two *Vitex agnus-castus *tablets or matching placebos were administered daily, in addition to three *Hypericum perforatum *tablets or matching placebos. The placebos were identical to the herbal tablets in size, colour, coating, weight and packaging. Placebo tablets comprised the excipients used in the active tablets; these were cellulose, modified starch, magnesium stearate and calcium hydrogen-phosphate. The daily dosage of the herbs was 1,000 mg *Vitex agnus-castus*, and 5,400 mg *Hypericum perforatum*. All tablets were manufactured under the Code of Good Manufacturing Practice by MediHerb Australia Pty Ltd.

### Participants

**Figure 2:**
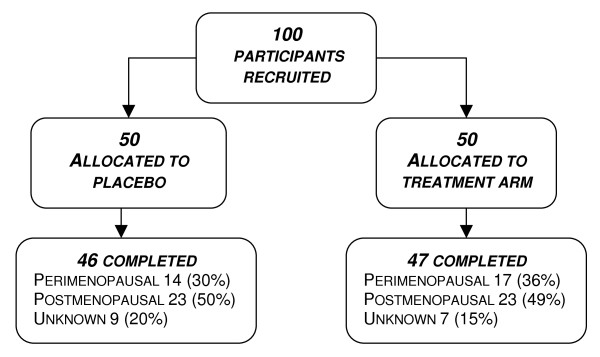
**Participant flow**.

Of the 93 women completing the trial, 47 had been randomised to the active treatment group and 46 to the placebo arm (see Figure 2). All were late-perimenopausal or postmenopausal women, aged 40 – 60 years. Details of the inclusion and exclusion criteria have been published previously [22]. Women were excluded if taking any medication known to interact with either study herb. Informed consent was obtained prior to study entry. Baseline visits were conducted in a clinic setting and follow-up contact by telephone. Medical clearance was obtained from a general practitioner prior to inclusion in the trial. Participants were requested to maintain their baseline dietary phytoestrogen intake during the trial.

**Figure 3:**
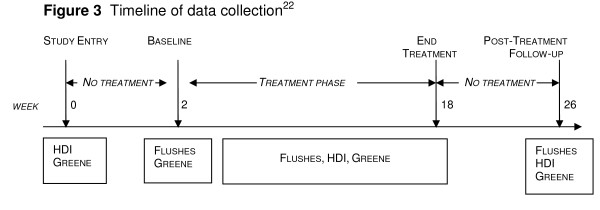
**Timeline of data collection^22^**.

Baseline data were collected for a range of variables, as previously published [22]. These were tested individually for their predictive ability. Measures administered at study entry, baseline and end of treatment phase are shown in Figure 3.

### Statistical Analyses

Data were analysed using Statistical Package for Social Science (SPSS) Version 16 with the assistance of a biostatistician. Data were analysed in two ways:

Firstly, focused tests of the difference between betas were conducted for each of the three individual endpoints. To do this, a series of hierarchical linear regression analyses were conducted using grouping as a dichotomous variable. An interaction variable was created for grouping × predictor for each potential predictor variable. The interaction of grouping and predictor was examined. Secondly, independent variables were assessed individually for their ability to predict the response in each arm on the three separate outcome measures. In each analysis, hierarchical regression was conducted in order to control for the baseline scores for the relevant outcome measures.

Response was defined as change in a favourable direction, that is, decrease in severity of symptoms. Because total GCS scores and GCS anxiety subscale scores were not independent, these were not entered simultaneously into a multiple regression analysis.

## Results

### Overall

The results of focused tests examining the interaction of group and predictor are presented in table 1. A significant difference was found in the predictive ability of anxiety at study entry between the two arms for all three endpoints, flushes *R*^2 ^= 0.41, Std. β = 0.43, *p *= 0.001; GCS *R*^2 ^= 0.29, Std. β = 0.45, *p *= 0.002; HDI-17 scale *R*^2 ^= 0.30, Std. β = 0.63, *p *< 0.001. Similarly, total GCS scores at study entry as a predictor of the subsequent response varied significantly between the two arms for all three endpoints of flushing, *R*^2 ^= 0.39, Std. β = -0.29, *p *= 0.012; overall menopausal symptoms measured on the GCS, *R*^2 ^= 0.31, Std. β = 0.43, *p *= 0.001; and depression measured on HDI-17, *R*^2 ^= 0.25, Std. β = 0.45, *p *= 0.001. Attitude to menopause was found to differ significantly between the two groups as a predictor of GCS scores, after controlling for baseline scores, *R*^2 ^= 0.27, Std. β = -0.34, *p *= 0.036.

**Table 1:** Differences between groups for predictors of week 16 endpoint scores, after controlling for baseline scores

*Interaction of Group and Predictor*	Completing Participants *n *= 93					
	
	*Flushes*		*Greene Climacteric Scale*		*HDI-17*	
	
	β (SE)	*p-*value	β (SE)	*p-*value	β (SE)	*p-*value
*GCS *score, study entry	0.78 (0.30)	0.012	0.87 (0.24)	0.001	0.65 (0.18)	0.001

*GCS *anxiety, study entry	2.14 (0.65)	0.001	1.71 (0.54)	0.002	1.75 (0.40)	<0.001

Attitude to menopause	-2.99(3.53)	0.40	-6.03(2.83)	0.036	-3.43(2.14)	0.113

Unstandardised β-coefficient (SE)

Results obtained from hierarchical linear regression analyses

### Individual Arms

Variables found to have significant predictive ability in the individual hierarchical linear regression analyses for any of the three endpoints for the individual arms are presented in Table 2. A negative β co-efficient indicates that more severe study entry scores were associated with milder symptoms at week 16. The overall change observed in GCS scores during non-treatment run-in was in the direction of *improvement *in symptoms.

**Table 2:** Predictors of week 16 endpoint scores for individual arms, after controlling for baseline scores

	Placebo Group *n *= 46						Active Treatment Group *n *= 47					
	
	*Flushes*		*Greene Climacteric* *Scale*		*HDI-17*		*Flushes*		*Greene* *Climacteric* *Scale*		*HDI-17*	
	
	β (SE)	*p-*value	β (SE)	*p-*value	β (SE)	*p-*value	β (SE)	*p-*value	β (SE)	*p-*value	β (SE)	*p-*value
Age at trial start	0.03 (0.28)	0.93	-0.26 (0.24)	0.28	-0.39 (0.17)	0.03	0.38 (0.32)	0.25	-0.21 (0.25)	0.41	0.23 (0.19)	0.24

Previous Herb Med effective	-10.9 (0.07)	0.63	-3.07 (1.84)	0.10	-3.14 (1.37)	0.03	-1.80 (3.04)	0.56	-4.46 (2.40)	0.07	-2.27 (1.88)	0.23

Attitude to menopause	-0.63 (2.18)	0.77	0.66 (1.88)	0.73	-0.24 (1.45)	0.87	-3.30 (2.97)	0.27	-4.54 (2.09)	0.04	-3.16 (1.60)	0.055

*GCS *score, study entry	-0.53 (0.17)	0.003	-0.43 (0.16)	0.012	-0.38 (0.13)	0.007	0.23 (0.26)	0.37	0.38 (0.26)	0.15	0.36 (0.18)	0.03

*GCS *anxiety, study entry	-0.97 (0.44)	0.03	-0.82 (0.39)	0.04	-0.33 (0.32)	<.001	1.16 (0.47)	0.016	0.65 (0.44)	0.14	0.68 (0.31)	0.02

Change in *GCS *scores during run-in	-0.02 (0.04)	0.59	-0.15 (0.06)	0.013	-0.07 (0.02)	0.005	-0.04 (0.08)	0.68	0.13 (0.08)	0.13	-0.07 (0.05)	0.15

Unstandardised β-coefficient (SE)

Results obtained from hierarchical linear regression analyses

For the individual arms, predictors of response identified, after controlling for baseline scores, were as follows.

### Anxiety at study entry

Anxiety at study entry significantly predicted *placebo *response on all the endpoints individually, with higher anxiety at entry associated with lower scores at end of treatment phase: flushes *R*^2 ^= 0.33, Std. β = -0.28, *p *= 0.03; GCS *R*^2 ^= 0.24, Std. β = -0.29, *p *= 0.04; HDI-17 scale *R*^2 ^= 0.34, Std. β = -0.66, *p *< 0.001. For the active treatment group, however, anxiety at study entry predicted *lack *of response for flushing, *R*^2 ^= 0.45, Std. β = 0.28 *p *= 0.02, and depression, measured on the HDI-17. *R*^2 ^= 0.28, Std. β = 0.31, *p *= 0.03.

None of the other predictors of the placebo response was relevant to the response in the active treatment arm (see Table 2).

In the active treatment group, positive attitude to menopause predicted response on GCS, *R*^2 ^= 0.34, Std. β = -0.27, *p *= 0.036.

### Baseline severity of scores

For the placebo group, Pearson's bivariate correlations revealed positive correlations between baseline scores and subsequent percentage improvement during the treatment phase for total GCS scores and anxiety subscale scores. No relationship between severity of scores and subsequent response was found for any of the endpoints for the active treatment group.

## Discussion

In the current study, there was a significant interaction between predictors of the response to placebo and the study intervention. Anxiety at study entry and overall menopausal symptoms at study entry (GCS scores) differed significantly between the two arms as predictors of the response on all three endpoints of flushing, overall menopausal symptoms and depression. Attitude to menopause differed significantly in its predictive ability between the two groups for the response on GCS scores. In terms of the individual arms, there were distinct differences in predictors of outcome observed between the placebo and active groups. Anxiety at study entry predicted *placebo *response for all the endpoints. In contrast, for flushing and depression in the treatment arm, study entry anxiety significantly predicted a *lack *of response to treatment, and had no effect on the Greene Climacteric scale scores. None of the other variables that predicted the placebo response was relevant to the treatment response. Improvement during non-treatment run-in predicted subsequent improvement during the treatment phase on GCS and HDI-17 depression scores for the placebo arm. This trend was not mirrored in the active treatment arm. For depression scores, older age at study entry predicted placebo response, as did prior positive experience with phytotherapy. However neither of these variables significantly impacted on outcomes in the active treatment arm. For the Greene Climacteric scale, baseline severity of symptoms was positively correlated with percentage improvement across the treatment phase in the placebo group, but not in the active treatment group.

Previous researchers of a range of other conditions have compared predictors of the responses in placebo and active arms within the same study where effects between the two arms differed [24-27]. Severity of symptoms at baseline has been found to differentially predict the placebo and treatment responses, with more severe depression being less responsive to placebo but more responsive to the pharmacological intervention [28]. Another study on acute bipolar manic episodes found symptom severity, age, number of previous hospitalisations to similarly predict the responses in both arms [25]. With regard to change in symptom severity during run-in, significant worsening of symptoms was associated with subsequent placebo response, but not "drug response" in an analysis of data from a functional dyspepsia study [29]. This contrasts with observations from the current study that *improvement *during run-in predicted subsequent response to placebo, but not to active treatment. However, to our knowledge, no previous studies have examined data from a RCT where superiority of 'active' treatment over placebo was not established to test the hypothesis that the predictors would be similar in the two arms.

In this study where efficacy of active and placebo were equivalent, the implications of the finding that the predictors of placebo response did not predict the treatment response are intriguing. As mentioned above, it has previously been suggested that the assumption of additivity of effects that underlies the practice of using placebos may not be a logical necessity [1]. It is possible that psychological mechanisms may operate in the placebo arm only in the absence of pharmacological effects, whereas effective interventions activate pharmacological mechanisms to the exclusion of psychological mechanisms [5]. Although it is generally accepted that there is a placebo component in the response to the active treatment when participants are blinded, the hypothesis of non-additivity implies that the pharmacological effects of an active intervention could override the psychologically-activated placebo component completely or partially. Essentially, the trial participants would experience either placebo or physiological intervention effects, but not both. If shown to be correct, this would invalidate the assumption that intervention effects are additive to placebo effects.

To our knowledge, no evidence exists from neurobiological studies of differential mechanisms operating in relation to menopausal symptoms, although there is some support for this phenomenon in relation to depression [6-8]. As depression, measured on the Hamilton Depression Inventory and the Greene Climacteric subscale, was one of endpoints of the current study, different mechanisms operating in the two arms in the current study cannot entirely be ruled out.

It is interesting to note that higher anxiety at study entry was a significant predictor of the placebo response, but predicted *lack *of response to active treatment. This supports the proposition that psychological factors are relevant to the placebo response, at least as moderators, if not mediators. The observation that improvement during non-treatment run-in predicted *placebo *response on two of the three endpoints, but did not predict *treatment *response, is consistent with the proposal that placebo-induced mechanisms, such as the release of endogenous opioids, may be activated in the anticipatory phase of the placebo response [15] and hence during therapist-patient or investigator-participant interaction [9]. The variance in the predictors of placebo and active response observed in the current study is consistent with the hypothesis that different underlying mechanisms may be operating in placebo and treatment arms.

Strengths of the study include the investigation of study entry scores (2 weeks prior to commencement of run-in), in preference to baseline scores, as potential predictors of the placebo response. The effect on psychological mechanisms of enrollment in a clinical trial would be expected to occur from the point of study entry, with the initiation of investigator-participant interaction and other context effects, rather than from initiation of the intervention [9]. However, because pharmacological effects of the intervention would only be observable from the point of administration of the intervention (see Figure 3), *baseline *scores were controlled for in the analysis.

A limitation in the interpretation of these findings is that there is no evidence, to our knowledge, supporting this phytotherapeutic combination as an effective treatment for menopausal symptoms. Therefore, a known pharmacological effect for this intervention in the treatment of menopausal symptoms has never been established. This study was a *post hoc *analysis of data from an RCT and as such, was not designed to explore neurobiological mechanisms. Thus, no definite conclusions can be drawn regarding any different mechanisms of action. Other possible limitations include the relatively small sample size, the use of exclusively subjective outcome measures, and the single scale of measurement for improvement during non-treatment run-in.

## Conclusion

In randomised, placebo-controlled clinical trials, greater understanding of the placebo response is needed to accurately dissect out placebo versus intervention effects. In order to conclude that a pharmacological intervention is ineffective if found *not *to be superior to placebo, it is essential to be confident that i) the placebo has no specific effect for the condition being examined, and ii) that the effects of the placebo and active intervention are completely additive, i.e. that subtracting the placebo effect from the treatment effect leaves the active intervention effect. The assumption of additivity has previously been questioned by other authors [1]. Early research on neuroanatomical and neurobiological mechanisms, primarily in the area of analgesia, suggests that placebo and pharmacological interventions may activate mutually exclusive pathways. The current findings could be explained in light of the theory of *non-*additivity. Further research is warranted into the neurophysiological basis of the placebo response to investigate the validity of the assumption of additivity. If this assumption were shown to be incorrect, it would have significant implications for the interpretation of results from placebo-controlled RCTs.

## Abbreviations

GCS: Greene Climacteric Scale; HDI-17: Hamilton Depression Inventory 17-item scale; SPSS: Statistical Package for Social Science

## Competing interests

Assoc. Prof. Kerry Bone was a co-founder of MediHerb, and is currently a consultant for MediHerb Australia Pty Ltd. He is related to Diana van Die (in-law). The other authors declare they have no competing interests.

## Authors' contributions

DVD, as principal investigator, had full access to all of the data in the study and takes full responsibility for the integrity of the data and the accuracy of the data analyses. DVD participated in the study concept and design, acquisition of data, analysis and interpretation of data, manuscript preparation and obtaining funding. HT participated in the study concept and design, study supervision, interpretation of data, critical revision of manuscript for important intellectual content and obtaining funding. HB participated in the study concept and design, study supervision, interpretation of data, critical revision of manuscript for important intellectual content. KB participated in the study concept and design, study supervision, interpretation of data, critical revision of manuscript for important intellectual content and obtaining funding. All authors read and approved the final manuscript.

## Pre-publication history

The pre-publication history for this paper can be accessed here:

http://www.biomedcentral.com/1471-2288/9/41/prepub
